# Assessment of the prevalence, serotype, and antibiotic resistance pattern of *Salmonella enterica* in integrated farming systems in the Maryland-DC area

**DOI:** 10.3389/fmicb.2023.1240458

**Published:** 2023-08-10

**Authors:** Zabdiel Alvarado-Martinez, Dita Julianingsih, Zajeba Tabashsum, Arpita Aditya, Chuan-Wei Tung, Anna Phung, Grace Suh, Katherine Hshieh, Matthew Wall, Sarika Kapadia, Christa Canagarajah, Saloni Maskey, George Sellers, Aaron Scriba, Debabrata Biswas

**Affiliations:** ^1^Biological Sciences Program, Molecular and Cellular Biology, University of Maryland, College Park, College Park, MD, United States; ^2^Department of Animal and Avian Sciences, University of Maryland, College Park, College Park, MD, United States; ^3^Department of Biology, University of Maryland, College Park, College Park, MD, United States

**Keywords:** *Salmonella enterica*, dairy farming, integrated crop-livestock systems, pathogen surveillance, antibiotic resistance

## Abstract

Implementation of organic/pasture farming practices has been increasing in the USA regardless of official certification. These practices have created an increasingly growing demand for marketing safe products which are produced through these systems. Products from these farming systems have been reported to be at greater risk of transmitting foodborne pathogens because of current trends in their practices. *Salmonella enterica* (SE) is a ubiquitous foodborne pathogen that remains a public health issue given its prevalence in various food products, but also in the environment and as part of the microbial flora of many domestic animals. Monitoring antibiotic resistance and identifying potential sources contamination are increasingly important given the growing trend of organic/pasture markets. This study aimed to quantify prevalence of SE at the pre- and post-harvest levels of various integrated farms and sites in Maryland-Washington D.C. area, as well as identify the most prevalent serovars and antibiotic resistance patterns. Samples from various elements within the farm environment were collected and screened for SE through culture and molecular techniques, which served to identify and serotype SE, using species and serovar-specific primers, while antibiotic resistance was evaluated using an antibiogram assay. Results showed a prevalence of 7.80% of SE pre-harvest and 1.91% post-harvest. These results also showed the main sources of contamination to be soil (2.17%), grass (1.28%), feces (1.42%) and unprocessed produce (1.48%). The most commonly identified serovar was Typhimurium (11.32%) at the pre-harvest level, while the only identified serovar from post-harvest samples was Montevideo (4.35%). With respect to antibiotic resistance, out of the 13 clinically relevant antibiotics tested, gentamycin and kanamycin were the most effective, demonstrating 78.93 and 76.40% of isolates, respectively, to be susceptible. However, ampicillin, amoxicillin and cephradine had the lowest number of susceptible isolates with them being 10.95, 12.36, and 9.83%, respectively. These results help inform farms striving to implement organic practices on how to produce safer products by recognizing areas that pose greater risks as potential sources of contamination, in addition to identifying serotypes of interest, while also showcasing the current state of antibiotic efficacy and how this can influence antibiotic resistance trends in the future.

## 1. Introduction

An increase in consumer demand for both plant and animal food products produced through organic/pasture farming has increased significantly as organic/pasture food producing methods are seen as a healthy and environmentally sustainable alternatives regardless of their certification status (Carlson et al., 2023). Over the last year, this industry has seen a nationwide increase in certified organic sales by 13% from 2019 to 2021, which accounts for $11.2 billion, 54% of which were mainly crops, while 46% were for livestock and related products (NASS, 2022). On the other hand, though many farms lack official certification accredited by relevant regulatory agencies to be properly labeled as organic, they still engage in practices that are compatible with organic farming, such as, limiting or eliminating the use of conventional antibiotics, synthetic antimicrobials, and disinfectants, having alternative animal housing spaces, utilizing natural fertilizers like manure and compost, and practicing animal/crop rotation (USDA National Organic Program, 2010). In keeping with the environmentally friendly approach to farming, numbers of organic/pasture farms grow both fresh produce and livestock in the same facility, commonly known as integrated crop-livestock farm (ICLF) system. This farming method is believed to increase production output and reduce land requirements for farming, while also recycling animal waste materials and repurposing it as a fertilizer for crops in the form of compost (Herrero and Thornton, 2001; Lemaire et al., 2014). Other farms, including dairy farms, have also been known to follow some of the practices laid out by regulatory agencies in order to comply with the organic farming standards. Products from these farming systems are often sold in local farmers markets, stores and roadside stands, with other producers making it to become providers for medium and large sized retail stores (Carlson et al., 2023). This fact has made some of these farms crucial part of the USA economy and supply chain for organic products, as they have become key providers of fresh organic produce, as well as organic meats and other animal products, while those that stay at the local level still manage to have a significant impact as the emergence of new registered farmers markets increase their reach across the entire country (King et al., 2010; Martinez et al., 2010). Considering the prevalence of organic products in the market, their impact on public health and food safety cannot be overlooked. Despite the enthusiasm surrounding organic farming for their progress in promoting sustainability, other findings suggest that the lack of antibiotic and synthetic antimicrobials use within these farms, the use of natural fertilizers like compost and manure, as well as increased contact and exposure to surrounding wildlife that might act as pathogen vectors, are factors that contribute to organic products being more at risk of being contaminated with common foodborne pathogens (Maffei et al., 2016). Further, there is an additional risk of cross-contamination brought about by produce being grown in proximity to different groups of live animals that often harbor pathogenic bacteria as part of their natural flora that can be transferred to food products, other live animals, handlers, and the surrounding environment (Park et al., 2012). On the other hand, other types of farms, such as organic dairy farms do not have to contend with the risk of contaminating produce, but there is still a significant concern for delivering products free from pathogens, while also properly managing animal waste and maintaining the health of the soil and surrounding environment (Lynch, 2022). Both dairy farm and ICLF systems face similar challenges when it comes to maintaining animal welfare and delivering safe products that are free of pathogens, but as the organic farming sector and organic farming practices grow to meet the demand of consumers, so do the risks of outbreaks, especially within markets consisting of limited agricultural space (Adl et al., 2011). In the Mid-Atlantic part of the USA there has been an increase in registered organic farms, (NASS, 2022), however, these numbers neglect the current number of farms that currently lack official organic certification, but are still implementing organic farming practices, or are in the process of transitioning from conventional farming to organic farming. Mid-Atlantic states like Maryland (MD) and Washington, District of Columbia (DC) have seen an increase in organic farming, officially totaling 120 certified farms, with 62 new farms being added as of 2022, all of which contributes to a sector of the local economy that amounts to >$50 million in sales (Maryland State Archives, 2023). Though most of these farms are family-owned businesses that mainly engage in local sales, the MD-DC area has also seen an increment in larger organic retail and wholesale stores, some of which are supplied by the local markets, and import from other states (Dimitri and Greene, 2002).

Considering the growing trend of organic/pasture integrated farming in the MD-DC area and the potential risks that might be involved with the products being produced in these, special attention must be given to specific foodborne pathogens. Among these foodborne pathogens, *Salmonella enterica* (SE) remains a major issue in the USA, as it is the bacterial pathogen responsible for the most amount of yearly cases of foodborne illness, accounting for more than 1.4 million diseases yearly (Scallan et al., 2011), as well as being responsible for a reported 153 single and multi-state outbreaks in 2021 that lead to 3679 illnesses and 768 hospitalizations (Centers for Disease Control and Prevention (CDC), 2021), while also accounting for 44% of outbreaks associate directly with organic products (Harvey et al., 2016). This increase in outbreaks can be attributed to the increase in consumption of these products but could also be associated to farming and processing practices at the pre-harvest and post-harvest levels that are unique to these farms (Iwu and Okoh, 2019; Sosnowski and Osek, 2021). When evaluating MD and DC, there were a reported 18 outbreaks that lead to 1786 illnesses and 450 hospitalizations (Centers for Disease Control and Prevention (CDC), 2021), though it is important to note that, despite improvements in surveillance and reporting, many sporadic outbreaks and illnesses go unreported (Zhang et al., 2022). The risk of SE is aggravated by their prevalence in products both at the pre-harvest and post-harvest levels of farming, particularly attributing them to contaminated fruits, vegetables, and poultry products (Interagency Food Safety Analytics Collaboration, 2022), but also isolating them from various environmental sources (Winfield and Groisman, 2003).

SE is a gram-negative, bacillus shaped facultative anaerobic bacteria that is comprised of over >2500 serotypes that have been identified, many of which can be associated with specific environments, foods, and animal hosts (Jajere, 2019). SE can be found ubiquitously across multiple environments, particularly in soil, water, and other surfaces (Bondo et al., 2016). The 5 most common serovars to be confirmed in cases of illness in the USA are Enteritidis, Newport, Typhimurium, Javiana and monophasic Typhimurium I 4,[5],12:i- (Centers for Disease Control and Prevention (CDC), 2018). The most common foods that have been attributed to causing illness with SE are chicken, produce, pork, beef, turkey, and eggs (Interagency Food Safety Analytics Collaboration, 2022). Some of these food groups have been associated with specific serovars in the USA, such as in the case of chicken being associated with SE Kentucky, beef being found to have Montevideo, turkey with Reading (USDA-FSIS, 2014). However, when looking at the prevalence of specific serotypes to an animal vector, Enteritidis and Typhimurium have been linked to chickens, while Newport and Typhimurium have been linked to cattle, Javiana being linked to equine and turkey sources, and monophasic Typhimurium I 4,[5],12:i- being found in chicken sources (Centers for Disease Control and Prevention (CDC), 2013). SE and many of its serovars can be found as part of the normal microflora of both wild and domestic animals without manifesting any disease or illness, making them vectors that can spread either through direct contact with the animal, through their products, or through their contact with the environment (Rukambile et al., 2019).

When considering the economic loss associated with the burden of SE illness directly associated with various food sources, the costs can amount to over $6.5 billion (Scharff, 2020). Given the importance of monitoring SE within smaller, local, organic farming operations, this study focuses on assessing the prevalence of SE across various farm elements and environments of both dairy farm and ICLF systems within the MD-DC area. After confirmation, SE isolates discovered in this study were serotyped and tested for resistance to various clinically relevant antibiotics. Co-resistance to specific antibiotics among the isolates confirmed in this study was also evaluated. The findings exposed in this research could lead to better facilities management practices at the pre-harvest and post-harvest levels of the local farming industry, as well as offer a detailed assessment of the prevalence of key SE serovars within the area of MD-DC.

## 2. Materials and methods

### 2.1. Sample collection by categories and sites

Both pre-harvest and post-harvest samples were collected from various sites within the MD-DC area in the USA, spanning 7 pre-harvest sites comprised of local farms that implement integrated farming practices, as well as other backyard producers, in addition to 3 post-harvest sites comprised of local markets. Farms were selected based on their location within the MD-DC area, as well as their type of farming practices that implement aspects of integrated farming regardless of official organic status certification, such as, but not limited to growing crops in proximity to animals for space maximization, avoidance of synthetic antimicrobials, antibiotics, pesticides and fertilizers in favor of natural fertilization through composing and manure processing, as well as allowing for pasture grazing, rotational grazing and open animal housing. Post-harvest sites were selected based on the same geographic criteria, while also being sites that are associated with the distribution of local produce and markets. A total of 3864 samples were collected (Table 1), with those in the pre-harvest category making up 3028 samples across multiple environmental categories, as well as unprocessed produce, while post-harvest samples made up 836 samples of multiple kinds of produce products. Pre-harvest samples included various elements from the farm environment, including animal feed, water, bedding, soil, grass, manure, and feces that had originated or had contact with poultry (both chicken and turkey), cattle, goat, sheep, and pigs, in addition to pre-processed produce spanning the categories of fruit, capsicum, vegetables, tubers, legumes, grains, leafy greens and herbs. Post-harvest samples were comprised of many kinds of fresh produce, including products within the categories of fruit, capsicum, vegetables, tubers, legumes, grains, leafy greens, and herbs. Sites were visited and sampled at least twice during the summer season (June–September) between the years of 2019 and 2021, in order to reduce seasonal variability and to comply with enough replicates between farms and sample types.

**Table 1:** List of sample types and categories collected from pre-harvest and post-harvest sites.

Sites sampled	Sample type	No. of samples per type	No. of samples per site
Pre-harvest*	Bedding	173	3,028
	Compost	219	
	Feces	370	
	Feed	275	
	Grass	269	
	Lagoon	51	
	Produce	874	
	Soil	461	
	Water	336	
Post-harvest**	Leafy greens	120	836
	Tubers	191	
	Fruits	162	
	Capsicum	154	
	Grains	40	
	Legumes	40	
	Herbs	90	
	Other	39	
Total			3,874

* Pre-harvest = 8; samples included environment, pre-harvest produce from various categories and animal samples including chicken, turkey, sheep, pig, and cattle.

** Post-harvest = 3; samples included post-harvest produce from various categories.

### 2.2. Sample processing, enrichment, selection, and growth conditions

All collected samples were placed in sterile plastic sample bags (VWR, PA, USA) and transported to the laboratory for immediate same-day processing. Sample processing was performed as described previously (Salaheen et al., 2015; Peng et al., 2016). Briefly, samples were suspended in sterilized 1 X phosphate buffered solution (PBS) (pH 7.2), while 1 mL of the resulting solution was used to inoculate 9 mL of Luria-Bertani (LB) broth (VWR, OH, USA) enriched with 10% sheep blood (Hemostat Laboratories, CA, USA). The enriched samples were incubated aerobically at 37°C for 24 h. After incubation, samples were streaked in *Salmonella-Shigella* (SS) agar (Difco-BD, MD, USA) and xylose lysine deoxycholate (XLD) agar (Criterion-Hardy Diagnostics, CA, USA) for selection and differentiation of *Salmonella* based on colony morphology (Nye et al., 2002). If present within the same sample, multiple colonies presumed to be *Salmonella* were selected for sub-culturing in LB agar for proper isolation and later used to prepare 20% glycerol stocks for each of the isolates.

### 2.3. Molecular confirmation, and serotyping of isolates

Molecular confirmation through species-specific and later serotype-specific primers (Table 2) was performed on presumptive positive isolates as described in previous studies (Peng et al., 2016). Briefly, isolates were re-grown in LB agar to isolate single colonies of the isolate. These were collected and resuspended in 1 x PBS to extract total DNA through heat lysis (95°C for 10 min). This solution was centrifuged, while the supernatant was collected and used as template DNA for confirmation and serotyping. Confirmation of SE was done using species-specific primers for genes *aceK* and *Salmonella oriC*, as described previously (O’Regan et al., 2008; Woods et al., 2008). Isolates confirmed to be SE were further serotyped using multiplex PCR with a combination of primers labeled STM 1–5, which are de-signed to identify the 30 most common SE serovars in the USA, by targeting and selectively amplifying specific regions within the bacterial chromosome depending on the serotype, as described previously (Kim et al., 2006). PCR amplification was performed following the manufacturer guidelines form the GoTaq^®^ Green Master Mix (Promega, WI, USA) with the following thermocycler protocol: 1 cycle at 94°C for 5 min, followed by 40 cycles of 94°C for 30 s, 59.8°C for 30 s and 72°C for 1 min, finishing with an ex-tension at 72°C for 5 min. These PCR products were visualized using a 2.5% (w/v) agarose gel prepared with 1 x Tris-Borate-EDTA (TBE) buffer and separated for 90 min at 100 V.

**Table 2:** Primers used for molecular confirmation and serotyping of SE.

Primer name	Primer sequence (5′-3′)		Product size (bp)	References
*aceK^+^*	F:	CCGCGCTGGTTGAGTGG	240	O’Regan et al., 2008
	R:	GCGGGGCGAATTTGTCTTTA		
*SalOriC*^+^	F:	GCGGTGGATTCTACTCAAC	461	Woods et al., 2008
	R:	AGAAGCGGAACTGAAAGGC		
STM-1*	F:	AACCGCTGCTTAATCCTGATGG	187	Kim et al., 2006
	R:	TGGCCCTGAGCCAGCTTTT		
STM-2*	F:	TCAAAATTACCGGGCGCA	171	
	R:	TTTTAAGACTACATACGCGCATGAA		
STM-3*	F:	TCCAGTATGAAACAGGCAACGTGT	137	
	R:	GCGACGCATTGTTCGATTGAT		
STM-4*	F:	TGGCGGCAGAAGCGATG	114	
	R:	CTTCATTCAGCAACTGACGCTGAG		
STM-5*	F:	TGGTCACCGCGCGTGAT	93	
	R:	CGAACGCCAGGTTCATTTGT		

^+^ Species-specific primers for confirmation of SE.

* Serotype-specific primers for identifying major SE serovars in the USA.

### 2.4. Determination of antibiotic resistance pattern of confirmed isolates

Antibiotic resistance was evaluated using an antibiogram assay, implementing 13 common antibiotics, and spanning 8 categories (Table 3). This assay was performed as has been describe before, with some modifications (Andrews, 2001). Briefly, several plates of Mullen-Hinton (MH) agar (Difco-BD, MD, USA), were independently prepared by combining a fixed volume of the molten agar with a specific concentration for a given individual antibiotic. Three concentrations were assessed for each antibiotic based on the Minimum Inhibitory concentration (MIC) breakpoints established by the Clinical & Laboratory Standards Institute (CLSI) guidelines manual (CLSI, 2023), which also served to provide the concentrations required to test for whether the isolates were susceptible, intermediate, or resistant to a given antibiotic. After solidifying, inoculation of the MH agar antibiogram plates was done with bacterial colonies that were previously sub-cultured and incubated overnight in LB broth without antibiotics at 37°C. After these isolates grew overnight, the bacterial suspensions were adjusted to an OD_600_ of 0.1, which was later used to inoculation the MH antibiotic agar plates with 2 μL for each isolate into their respective quadrants. After inoculation, MH antibiotic agar plates were also incubated at 37°C overnight for later recording growth pattern of each isolate in the respective MH antibiotic agar plate and compared to the standard breakpoints established by the CLSI guidelines manual for Enterobacterales, which includes standards for bacteria in the *Salmonella* genus.

**Table 3:** Antibiotic used for antibiogram assay with corresponding breakpoints used to test tolerance of isolates.

Antibiotic category	Antibiotic	Breakpoint concentrations (μg/mL)*		
		Susceptible	Intermediate	Resistant
Penicillins	Ampicillin	8	16	32
	Amoxicillin	8	16	32
Macrolides	Azithromycin	16	24	32
Cephalosporins	Cephradine	4	8	16
	Ceftriaxone	1	2	4
Phenolics	Chloramphenicol	8	16	32
Quinolones	Ciprofloxacin	0.06	0.12	1
Aminoglycoside	Gentamycin	4	8	16
	Kanamycin	16	32	64
	Streptomycin	16	24	32
Tetracyclines	Tetracycline	4	8	16
	Oxytetracycline	4	8	16
Folate pathway inhibitors	Trimethoprim-sulfamethoxazole	2–38	3–57	4–76

* Antibiotic breakpoints were selected based on the standards established by the CLSI manual.

Further analysis to determine antibiotic co-resistance of isolates was performed by calculating the frequency of isolates that were resistant to two antibiotics. Performing this calculation for every possible antibiotic pair allowed for the determination of co-resistance frequency. These values were represented in a chord plot, using the individual antibiotics as nodes, the existence of a co-resistant isolate forming the edge between nodes, and the weight of these edges being determined by the frequency of isolates sharing the same co-resistance pattern. The data was visualized using the Holoviews package for Python.^1^

### 2.5. Statistical analysis

Data analysis was performed using the Chi-square statistical analysis to determine significant association between the number of samples positive for SE and the multiple sample categories that were collected for the study (Singhal and Rana, 2015; Barceló, 2018). This test was also later used to determine if there was a significant association regarding the identification of specific SE serotypes from confirmed samples, as well as to analyze their antibiotic resistance pattern. Additional calculations for standard residual values were performed to determine the categories that contributed to driving significance.

## 3. Results

### 3.1. Prevalence of *Salmonella enterica* (SE) across collection sites and respective categories

**Figure 1:**
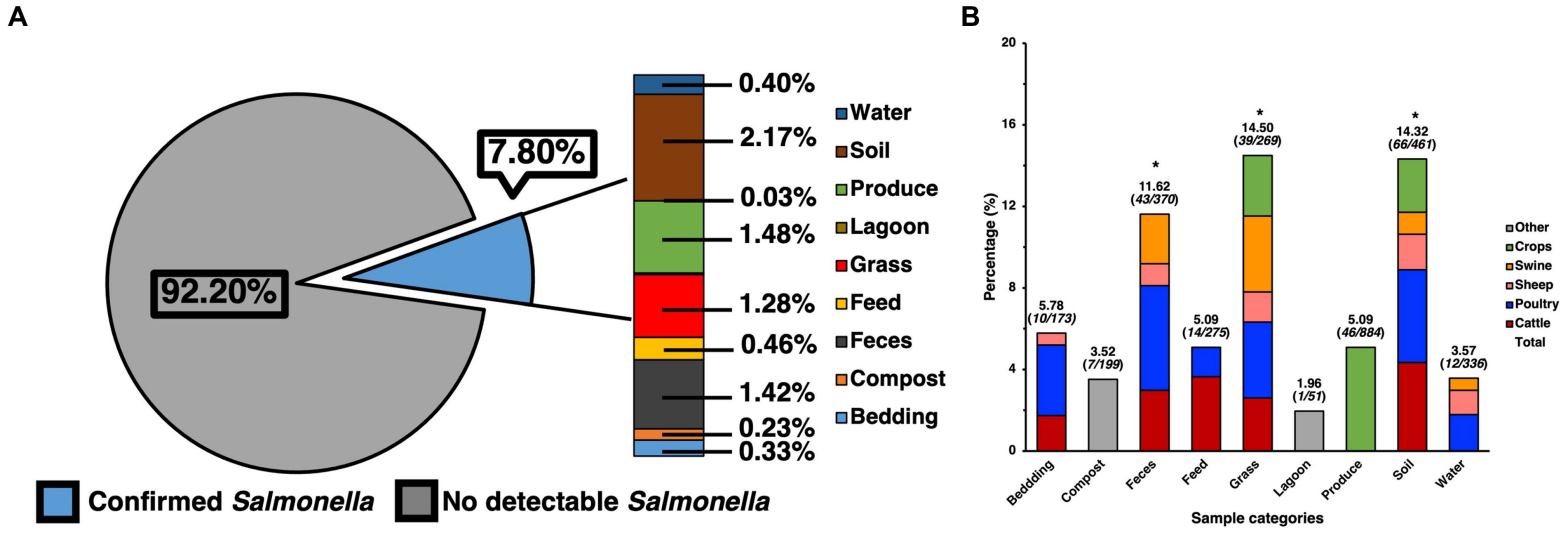
Prevalence of SE isolated and confirmed from various samples taken from various farm elements of pre-harvest farm environments and their distribution. **(A)** Prevalence of SE by category and their contribution to total prevalence of pre-harvest samples. **(B)** Ecological distribution of SE within categories based on positivity of SE for the specific category and sample origin. Chi-square analysis was used, along with standard residual analysis and statistical significance (*p* < 0.05) has been denoted by a star a (*).

The ecological prevalence of SE within environmental elements and products from the farms that were sampled pre-harvest, as well as post-harvest was assessed through selective and differential culture methods but was later molecularly confirmed (Figure 1). A total of 2237 presumptive SE colonies were collected from the culture plates, spanning 1004 of the samples, as multiple colonies were collected from the plate. After molecular confirmation, it was revealed that 500 of these were confirmed as SE, while at the pre-harvest level, out of 3038 samples a total of 232 samples were found to be positive for SE after molecular confirmation, resulting in a total prevalence of 7.80% (Figure 1A), which was later analyzed by preparing a contingency table that allowed for the use of a Chi-square test for the full data set, showing these results to be significant (*p* < 0.05) and not due to chance within the environmental samples. When specifically analyzing the ecological distribution of SE among the environmental samples collected from the pre-harvest sites, soil was found to be the greatest contributors to the total positivity rate (2.17%), feces (1.42%) and grass (1.28%), which were confirmed as being the major categories driving significance after calculating the standard residual value and finding these to have the largest residuals. Though produce had a numerically similar prevalence of SE compared to other categories (1.48%), the resulting standard residual did not show this category to be a statistically significant contributor to overall prevalence. Further analysis of SE positivity within each of the specific sample categories collected for this study (Figure 1B) elucidated more information regarding SE distribution within soil (14.32%, 66/461), feces (11.62%, 43/370), grass (14.50%, 39/269), and produce (5.15%, 45/874), feed (5.09%, 14/275), water (3.57%, 12/336), bedding (5.78%, 10/173), compost (3.52%, 7/199), and lagoon water (1.96%, 1/51). Further analyses were performed to determine the major animal contributors to the categories with the most significant prevalence. These revealed cattle, poultry, and swine to be the most significant (*p* < 0.05) contributors to SE positivity in feces and grass, while cattle and poultry were the most significant contributors in soil.

**Figure 2:**
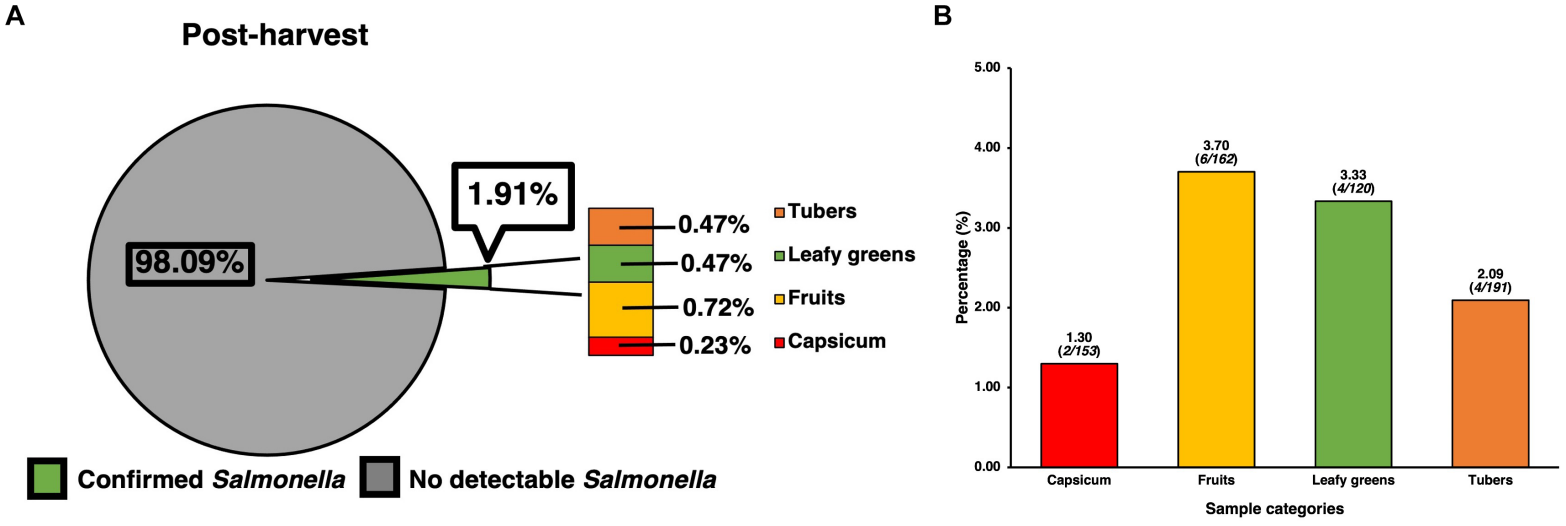
Prevalence of SE isolated and confirmed from various post-harvest produce samples and their distribution. **(A)** Prevalence of SE by produce category and their contribution to total prevalence of post-harvest samples. **(B)** Distribution of SE within common produce categories based on the positivity of SE for the specific category. Chi-square analysis was used, along with standard residual analysis and statistical significance (*p* < 0.05) has been denoted by a star a (*).

Prevalence of SE for samples collected at the post-harvest level was found to contain 16 confirmed positives for SE out of 836 samples, resulting in a total prevalence of 1.91% (Figure 2A), though analysis using the chi-square test revealed these to be only numerical and not statistically significant. However, when compared to the pre-harvest produce category alone, post-harvest produce showed a statistically significantly (*p* < 0.05) lower prevalence than pre-harvest (5.09%). In terms of the distribution of SE across sample types, since all post-harvest samples were produce/vegetable, further itemization was done based on the most used classifications for the different kinds of produce samples that were collected. Though many kinds of fresh produce were collected, processed, and assessed for presence of SE, the ones found to be positive were those within the larger categories of fruit (0.72%,), tubers (0.47%), leafy greens (0.47%) and capsicum (0.23%), with standard residual analysis showing fruits and leafy greens having stronger associations. The prevalence of SE within each individual category (Figure 2B) were fruits (3.70%, 6/162), tubers (2.09%, 4/191), leafy greens (3.33%, 4/120), and capsicum (1.30%, 2/153).

### 3.2. Major SE serotypes identified among confirmed isolates

**Figure 3:**
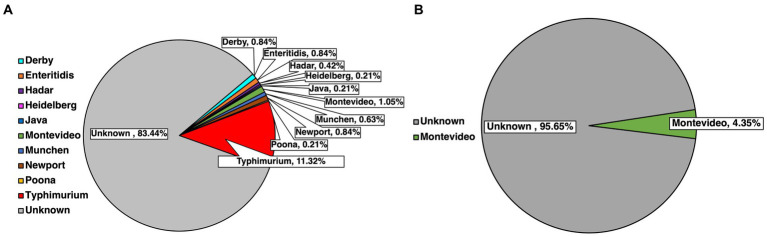
Identification of common SE serotypes within confirmed isolates from pre-harvest **(A)** and post-harvest **(B)** samples.

Serotyping for the top 30 major SE serotypes found in the USA was done for every isolate previously confirmed molecularly by using species-specific primers. Serotype identification was done based on select amplification, subsequent separation and visualization of primers labeled STM 1–5 (Figure 3). A total of 500 isolates were serotyped, with 477 of these being pre-harvest and 23 being post-harvest, while some were bacteria initially isolated form the same sample. Of the pre-harvest samples, 10 serotypes were identified, namely from most to least numerically prevalent, Typhimurium (11.32%, 54/477), Montevideo (1.05%, 5/477), Derby (0.84%, 4/477), Enteritidis (0.84%, 4/477), Newport (0.84%, 4/477), Munchen (0.63%, 3/477), Hadar (0.42%, 2/477), Heidelberg (0.21%, 1/477), Java (0.21%, 1/477) and Poona (0.21%, 1/477), with 83.44% (398/477) remaining unserotyped, that is the primer combination that were amplified had no match to the serotypes being tested for. Within the post-harvest category, the serotype that was detected was Montevideo (4.35%, 1/23), while the remaining samples remained unserotyped (95.65%, 22/23).

### 3.3. Antibiotic resistance patter of isolates against major antibiotics

**Figure 4:**
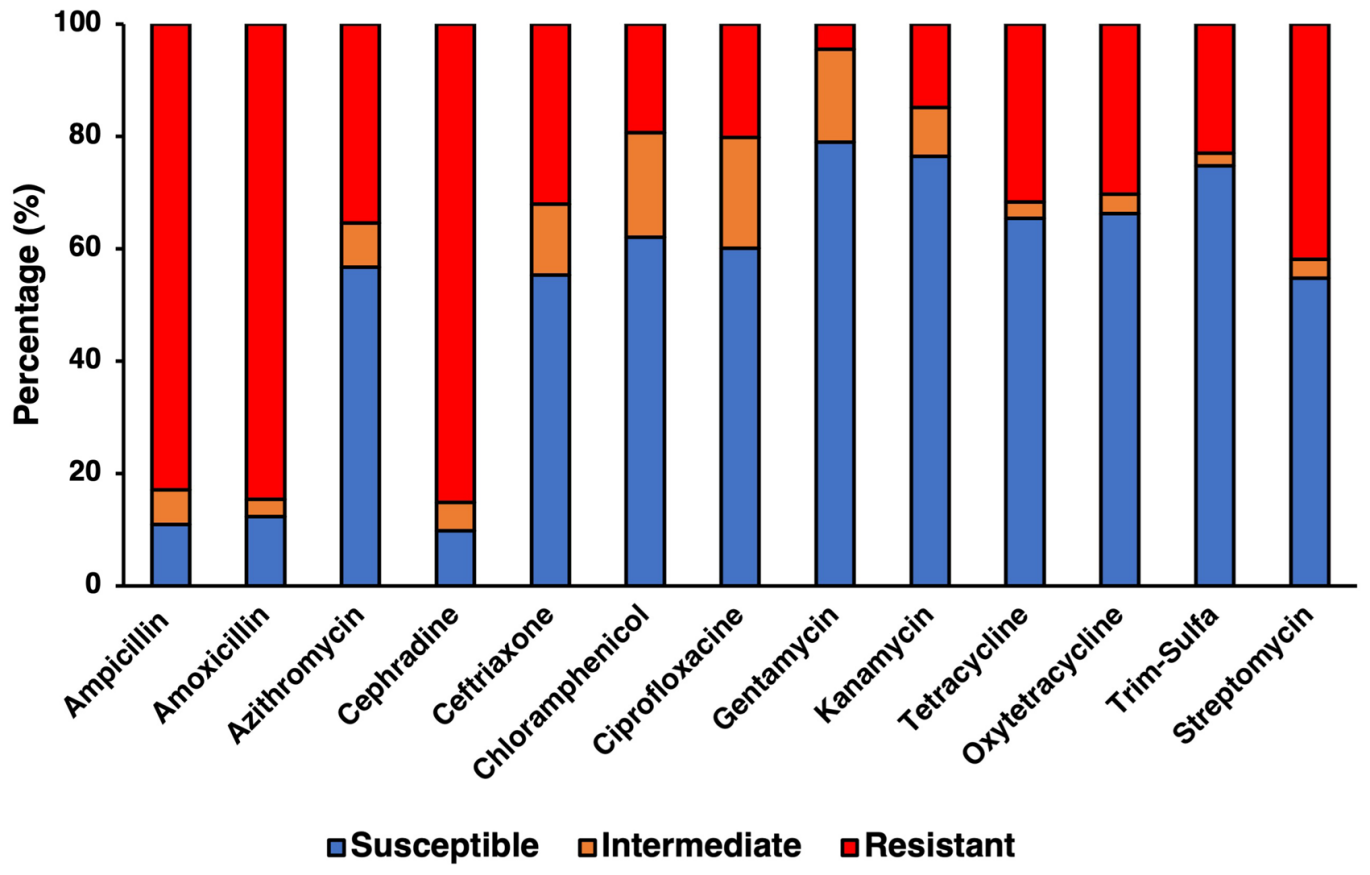
Antibiotic resistance profile for confirmed SE isolates determined from antibiogram assay testing for 13 antibiotics using concentrations previously determined to be the breakpoints for susceptible, intermediate, and resistant for *Salmonella*.

Resistance to specific antibiotics was assessed through an antibiogram assay, in which three concentrations of 13 antibiotics spanning 8 categories (Table 3) where evaluated for their capability to inhibit the growth of the confirmed farm isolates (Figure 4). Concentrations used for each antibiotic in the antibiogram assay were determined using the guidelines from the CLSI manual. Using multiple concentrations allowed for determining the level of tolerance for each of the isolates to a specific antibiotic, leading to categorizing isolates as either being susceptible, intermediately resistant, or resistant to the antibiotics tested, based on the concentration that exhibited growth after incubating for 24 h. The antibiotics to which most isolates were susceptible based on their respective breakpoints were to aminoglycosides like gentamycin (78.93%, 281/356) and kanamycin (76.40%, 272/356), however streptomycin was significantly less effective (54.78%, 195/356). Antibiotics that had a similar percentage of susceptible isolates were the tetracyclines like tetracycline (65.45%, 233/356) and oxytetracycline (66.29%, 236/356), as well as the folate pathway inhibitor mix of trimethoprim-sulfamethoxazole (74.72%, 266/356), with the remaining percentage of isolates being mostly resistant. Slightly more isolates were resistant to the phenolic and quinolone antibiotics tested, namely, chloramphenicol and ciprofloxacin, which still had a majority susceptible isolates (62.08%, 221/356 and 60.11%, 214/356, respectively). Cephalosporines showed to be less effective than other antibiotics, as shown in the samples susceptible to ceftriaxone being only slightly higher than half (55.34%, 197/356), while on the other hand the majority of isolates were resistant to cephradine (82.58%, 303/356). The macrolide, azithromycin had slightly more than half of isolates being susceptible (56.74%, 202/356). As a category, penicillin were the antibiotic group with the most rate of resistance, with amoxicillin and ampicillin having the greatest number of resistant isolates (84.55%, 301/356 and 82.87%, 295/356, respectively). Statistical analysis of the growth pattern exhibited in the antibiogram that there was a statistically significant higher frequency of susceptible isolates, than intermediate or resistant ones, however gentamycin and kanamycin were found to be the main contributors to susceptibility.

### 3.4. Chord analysis for determining co-occurrence of antibiotic resistance among isolates

**Figure 5:**
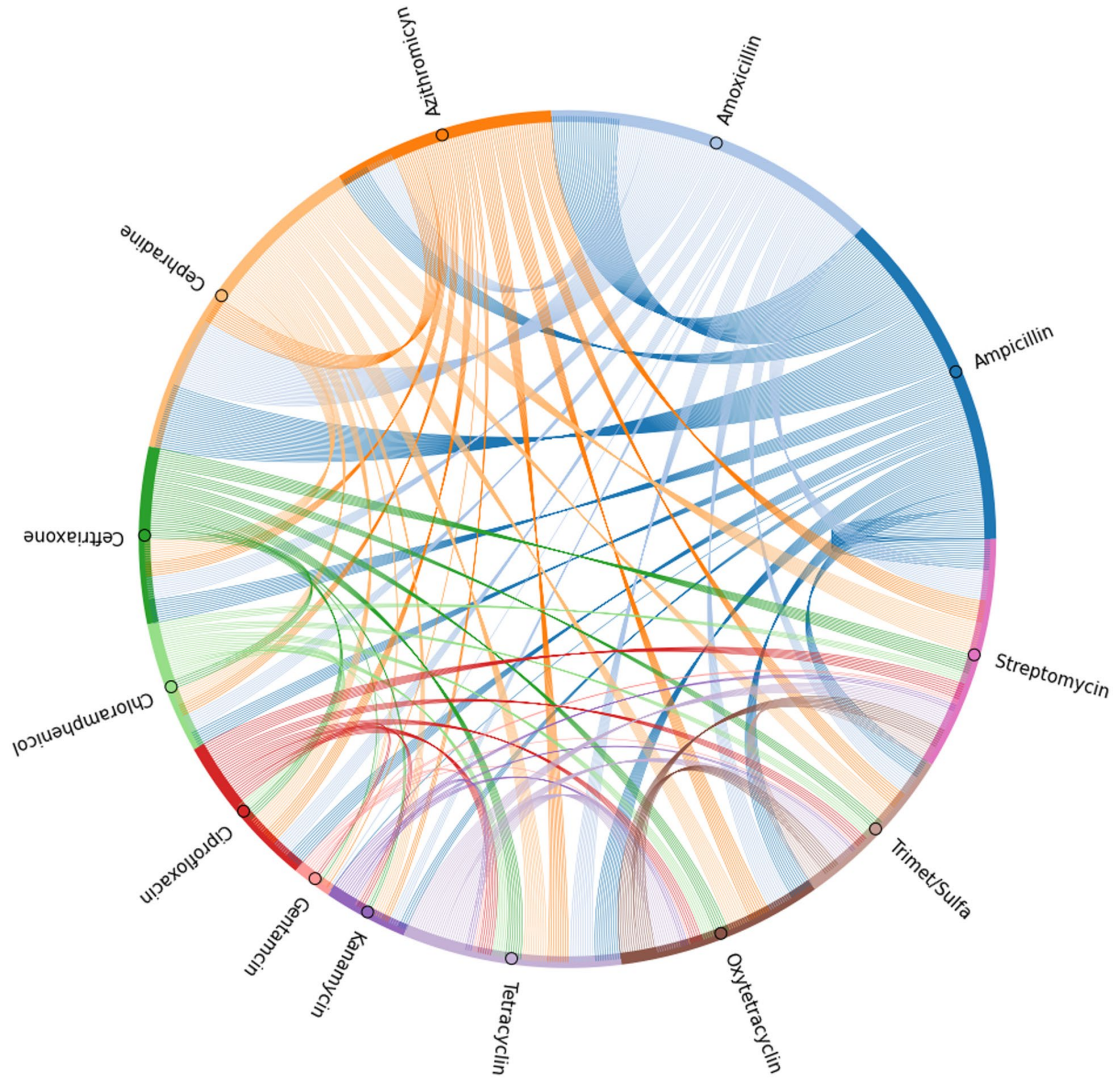
Chord plot generated based on the incidence of co-resistance to antibiotic pairs among the isolates tested in the antibiogram.

To further elucidate the incidence of co-resistance to specific antibiotic pairs within the SE isolates, the number of isolates found to be resistant to the same pair of antibiotics was recorded for every possible antibiotic pair (Supplementary Table 1). These values were used to generate a chord plot for further visualization of the incidence and relationship between resistance to all antibiotic pairs (Figure 5). According to the calculations for incidences of co-resistant isolates, the pairs with the highest amount of co-resistant isolates were amoxicillin-cephradine (281), followed by ampicillin-amoxicillin (278) and ampicillin-cephradine (268), showing penicillin and cephalosporines to have the most cases of co-resistance by antibiotic type. When comparing penicillin to other groups, there were less co-resistant isolates, but notable values were seen for ampicillin-streptomycin (132), amoxicillin-streptomycin (122), amoxicillin-azithromycin (112) and ampicillin-tetracycline (103). Other notable pairs were cephradine-streptomycin (127) and cephradine-azithromycin (112). On the other hand, the best pairings were those with gentamycin, showing the lowest instance of co-resistance, beginning with gentamycin-chloramphenicol (3), followed by gentamycin-trimethoprim-sulfamethoxazole (5), and even including pairings with panicillins like in the case of gentamycin-amoxicillin (8) and gentamycin-ampicillin (9). Other antibiotic pairs that had lower incidences of co-resistance were kanamycin-gentamycin (10), kanamycin-chloramphenicol (10), gentamycin-ceftriaxone (10), gentamycin-azithromycin (10), and gentamycin-oxytetracycline (10).

## 4. Discussion

Surveillance of SE in pasture farms specifically backyard and integrated farms withing the Mid-Atlantic region of the USA is merited given the rapid growth of this market in this region of the country (NASS, 2022). As a ubiquitous foodborne pathogen, SE continues to be a burden on the USA healthcare system, as well as an economic burden for food industries and consumers, which could hinder the profitability and viability of smaller producers using alternative farming systems (Ollinger and Houser, 2020). Previous studies have evaluated the prevalence of SE in organic meats, particularly poultry, generated from farms within the MD-DC area (Peng et al., 2016). This study assessed various environmental elements within farms that could be potential sources of SE and serve as transmission pathways, as well as produce samples collected from farms (pre-harvest) and on-farm markets or farmers markets (post-harvest), while also identifying various serotypes of this pathogen in multiple isolates that were previously confirmed to be SE. Compared to the findings of previous studies, there was a lower overall prevalence of SE detected in in the current study. Though environmental factors play an important role SE contamination, meat products are at a particularly higher risk of contamination, post-harvest, due to there being at a higher risk of cross-contamination during processing, as there is potential contact with contaminated equipment and other animal body parts (Golden et al., 2021).

In the current study, notable sources of SE were, soil and grass, especially form fields used for grazing and pasturing cattle and poultry. These results are compatible with previous meta-analyses that have shown soil to be a major reservoir for many common foodborne pathogens, especially SE, as it has been found to survive in this environment for extended periods of time (Wang et al., 2023). Once in soil, SE can spread to other fomites, such as grass, which was observed in the current study, but it is important to note that it could also spread to nearby water sources and crop-fields in the form of runoff. Another major source of SE was fecal matter form various animals, primarily from poultry, cattle and swine, which could be an explanation for the prevalence of the pathogen in soil, as it gets seeded with the bacteria as animals graze, while the soil conversely serves as a source of re-inoculation, as well as cross-contamination for other animals that are rotated to graze on the same land (McAllister and Topp, 2012; Joseph et al., 2021). Many integrate/pasture farms engage in animal rotation, increasing the chances of cross-contamination between the animals that graze on the same plots of land even if the animals are not there simultaneously, which could serve as an explanation as to why chicken and cattle samples were highly positive, since these were two animal groups that were often rotated between the same pasture sites across the farm. Various waste management strategies like composting could also be potential sources of contamination, as studies have shown that when done incorrectly pathogenic bacteria can survive the composing process, making this a potential source of contamination, particularly for plant products and to surrounding soil (Gong et al., 2005; Brinton et al., 2009).

Identifying these sources of SE within alternative and fast-growing farming systems is an important step for generating safer products, but also reducing the negative impact that these farms could pose to the surrounding environment, which often includes other farms, important natural resources, as well as commercial and residential areas (Hudson and Soar, 2023; Wu et al., 2023). On the other hand, when comparing pre-harvest with post-harvest produce, there was a statistically significantly higher prevalence in the pre-harvest samples. This could be attributed to the exposure that these have to the surrounding environment, as well as the lack of processing and screening that these products go through before being sold. However, the prevalence of SE in the post-harvest samples could still be attributed to specific challenges associated with produce processing, in which contamination can occur during the transportation, management, and even washing of the product with reused water (Rahman et al., 2022).

SE is known to have over 2500 serovars, many of which can be attributed to outbreaks within the USA (Andino and Hanning, 2015). Serotyping of the confirmed isolates from this study revealed SE serotype Typhimurium to be the most prevalent out of the isolates that wee serotypeable. This serotype was also found only in pre-harvest samples. *S. typhimurium* is known to be a widely spread serotype, spanning multiple environments and sources, in addition to being associated to multiple vegetable and animal products, including poultry, cattle and swine, while also being capable of causing disease (Ferrari et al., 2019). The next most prevalent serotype was Montevideo, which has been strongly correlated in the past with beef products in North America, as well as with some produce products (Andino and Hanning, 2015). Other serovars that were identified were SE serotypes Derby and Enteritidis, which are also categorized among some of the most common serotypes in the USA. Though previous researchers have been able to attribute the serotype Enteritidis with more outbreaks, the food sources with which it is associated with are narrower, while serotypes like *S. typhimurium* span multiple sources (Jackson et al., 2013). The current study did not find statistically significant association of a given serotype to a specific sample category, however it is important to note that, though Typhimurium was found in samples associated with poultry, cattle, swine, soil and vegetables, other serotypes like Enteritidis, Newport and Derby were mostly found in poultry, while Hadar was only found in cattle, and Montevideo in both cattle and vegetables. Another aspect of the current study was the fact that the majority of isolates for both pre- and post-harvest categories remained unserotyped as they could not be categorized within the 30 most common serotypes found in the USA given their gene expression of the multiplex technique that was used. This lack of identification for the current isolates could account for the lack of statistical significance associated with the isolates that were serotyped. Though multiplex PCR techniques have been used in the past with high accuracy and cover identification many common serovars (Shi et al., 2015), there are still limitation regarding mutations in the bacteria that might affect primer specificity, which is also a contributing factor to many outbreaks not being associated with specific serotypes (Chanamé Pinedo et al., 2022). Another possibility is that the unserotyped isolates belong to serovars other than the main 30 clinically relevant ones that the multiplex PCR assay was designed to detect, which could include other less studied subspecies of SE that might have a lower pathogenicity to humans or are more commonly found in animals as pathogens. Previous research has reported some of these subspecies to also be highly prevalent in farm environments and in some animals, both wild and domestic, and account for a significant percentage of isolates found in some farms (Lamas et al., 2018). Further studies involving the use of whole genome sequencing and *in silico* methods to identify specific genes in these isolates that can reveal their serotype or identify mutations in the genes that were tested that could have led to the primers not being able to properly express (Diep et al., 2019).

Antibiotic resistance of SE isolates was tested for 13 clinically relevant antibiotics, however it is important to note that resistance to some antibiotics can also translate to resistance to others, depending on the mechanism conferring the resistance, as well as the genes involved (Nikaido, 2009). In addition to the intrinsic antibiotic resistance mechanisms that bacteria carry, other antibiotic resistance genes can be transferred horizontally from resistant bacteria to non-resistant ones, including from non-pathogenic to pathogenic bacteria (Dionisio et al., 2023). This dynamic is especially prevalent in the soil, where there is a complex microflora comprised of bacteria, archaea, fungi, and viruses, as well as conglomerate of genes known as the resistome, all of which contribute to bacteria being able to integrate additional antibiotic resistance genes and become increasingly resistant (Walsh, 2013; Von Wintersdorff et al., 2016). In addition to this, the environment also serves as a pressure that helps select for increasingly resistant microbes and in the case of an area that has trace amounts of commonly used antibiotics, these will also serve as a selective force. The presence of trace amounts of antibiotic in soil can be linked to the overuse of these compounds in healthcare and animal production, with animal farming being of note because of their use as sub-therapeutics, therapeutics, and growth promoters (Chokshi et al., 2019). This created a cycle where trace amounts of antibiotics reached multiple sources in the environment, as well as to other animals, animal products and human consumers (Woolhouse and Ward, 2013). Though many regulatory measures have been put into place for reducing antibiotic resistance in the environment, tackling this issue remains a challenge (Mann et al., 2021). Reducing use of antibiotics has shown to reduce the incidence of antibiotic resistant bacteria (Tang et al., 2017), and in the case of organic farms, they do not use antibiotics in their production practices. Though previous research would suggest that these factors would make organic farms free of antibiotic resistant bacteria, recent findings have shown that there was no significant difference in the presence of antibiotic resistance genes between conventional and long-standing organic farms, though some bacterial isolates were more resistant to a specific type of antibiotic (Sancheza et al., 2016; Armalytė et al., 2019). These findings suggest that regardless of the time that a farm has been engaging in organic practices, it could still be at risk, as it could have trace amounts of antibiotics and other synthetic antimicrobials in their soil, while also having a resistome, and pre-existing bacteria that could potentially harbor antibiotic resistance. In the current study, a significant number of SE isolates showed resistance to each antibiotic, but further genomic analysis would be required to determine the origin of this antibiotic resistance and the genes involved. However, with the exception of ampicillin, amoxicillin and cephradine, all other antibiotics showed that at least more than half of the isolates were susceptible to them.

When analyzing the antibiotic resistance pattern of the isolates form the current study through testing the MIC at various breakpoints for each antibiotic, there were specific groups of antibiotics that were more effective than others. Of all the antibiotic categories tested, penicillins, namely ampicillin and amoxicillin, as well as the cephalosporine, cephradine, were the least effective antibiotics, as most of the isolates were resistant to them. These findings are in accordance with previous groups that have shown a high prevalence of resistance to penicillins in various SE serotypes, but in addition to that, the chord analysis in the current study also showed a high level of co-resistance between ampicillin, amoxicillin and cephradine, which previous researchers have found to be correlated, as they share similar mechanisms of action through the β-lactam site (Nair et al., 2018). Though previous research has identified genes associated with resistance to aminoglycosides like gentamycin, kanamycin and streptomycin, as well as folate pathway inhibitors like the trimethoprim-sulfamethoxazole, these antibiotics are still often prescribed as second line antibiotics in clinical cases (Frye and Jackson, 2013), and the results from the antibiogram of the current study showed gentamycin, kanamycin and a combination of trimethoprim-sulfamethoxazole to be the most effective against most isolates, while streptomycin was not as effective. In terms of co-resistance, the antibiotic pairs with the lowest occurrence were gentamycin-chloramphenicol, gentamycin-trimethoprim-sulfamethoxazole, gentamycin-ciprofloxacin, and even gentamycin-ampicillin and -amoxicillin. This could be attributed to the individual effect of gentamycin, relative to the efficacy of the other antibiotics, as these were not tested simultaneously, as to be able to properly determine any synergistic activity. However, in the past, aminoglycosides have been proposed as potential antibiotics that could be paired with others to achieve a synergistic effect (Umemura et al., 2022). In addition to synergistic mechanisms of action, using antibiotics that require the bacteria to employ multiple mechanisms of resistance could be another approach to antibiotic selection for clinical uses (Kakoullis et al., 2021). Another two groups of antibiotics that are important to mention from this study are tetracycline and ciprofloxacin, as when these were compared to the national average reported by the National Antimicrobial Resistance Monitoring System (NARMS) for the percentage of resistant SE, they were more susceptible bacteria than in NARMS reports, which is important since these are often times used as indicators for multidrug resistance and cases of co-resistance in SE and other coliforms (Hopkins et al., 2005; Food and Drug Administration (FDA), 2022). Though it remains unclear whether antibiotic resistant SE translates to more or less virulent bacteria, recent research has found correlations between specific genes related to virulence and multi-drug resistance, warranting further study into the current effective antibiotic treatments that are available to deliver more accurate and safer treatments (Higgins et al., 2020).

## 5. Conclusion

Surveillance and risk assessment remain crucial tools for preventing outbreaks and producing safer food, especially in small and medium sized producers within the organic/pasture farming systems. SE remains prevalent in the farm environment, especially within the soil, which serves as a reservoir for the pathogen. The SE serovar Typhimurium remains of the most prevalent serotype, which coincides with the previous knowledge that Typhimurium us ubiquitous across multiple environments and is attributable to multiple animals. This will provide valuable information for tracing and containing outbreaks. On the other hand, the current antibiotic pattern exhibited by the isolates of this study show that, despite the absence of antibiotic use in these farming systems, there is still the presence of antibiotic resistant bacteria. However, some antibiotics were more effective than others, which can be used in the future to further study the mechanisms behind antibiotic resistance in farming environments, while also inform future treatment protocols for clinical cases of SE infections.

## Data availability statement

The original contributions presented in the study are included in the article/Supplementary material, further inquiries can be directed to the corresponding author.

## Author contributions

DB: conceptualization, supervision, project administration, and funding acquisition. ZA-M and DB: methodology and writing—review and editing. ZA-M and DJ: formal analysis. ZA-M, ZT, AA, C-WT, AP, GSu, and MW: sample acquisition, preparation, and processing. ZA-M, DJ, AP, GSu, KH, MW, SK, CC, SM, GSe, and AS: execution of experiments. ZA-M: writing—original draft preparation. All authors have read and agreed to the published version of the manuscript.

## Funding

This research was funded by the United States Department of Agriculture (USDA), National Institute of Food and Agriculture (NIFA) (grant no. 20185110628809).

## Conflict of interest

The authors declare that the research was conducted in the absence of any commercial or financial relationships that could be construed as a potential conflict of interest.

## Publisher’s note

All claims expressed in this article are solely those of the authors and do not necessarily represent those of their affiliated organizations, or those of the publisher, the editors and the reviewers. Any product that may be evaluated in this article, or claim that may be made by its manufacturer, is not guaranteed or endorsed by the publisher.

## Footnotes

^1^https://holoviews.org/
