# Supplementary material for: Assessment of the prevalence, serotype, and antibiotic resistance pattern of Salmonella enterica in integrated farming systems in the Maryland-DC area
Source: Front Microbiol. 2023 Aug 10;14:1240458. doi: 10.3389/fmicb.2023.1240458 (PMC10448900; doi:10.3389/fmicb.2023.1240458)
Supplement: Supplementary file 1 [file Table_1.docx]

Supplementary Material

Assessment of the prevalence, serotype, and antibiotic resistance pattern of *Salmonella enterica* in integrated farming systems in the Maryland-DC area

Zabdiel Alvarado-Martinez^1†^, Dita Julianingsih^2†^, Zajeba Tabashsum^1^, Arpita Aditya^2^, Chuan-Wei Tung^2^, Anna Phung^3^, Grace Suh^3^, Katherine Hshieh^3^, Matthew Wall^3^, Sarika Kapadia^3^, Christa Canagarajah^3^, George Sellers^3^, DebabrataBiswas^1,2,4*^

^1^Biological Sciences Program-Molecular and Cellular Biology, University of Maryland-College Park, College Park, 20742, MD, USA

^2^Department of Animal and Avian Sciences, University of Maryland-College Park, College Park, 20742, MD, USA

^3^Department of Biology, University of Maryland-College Park, College Park, 20742, MD, USA

^4^Center for Food Safety and Security Systems, University of Maryland-College Park, College Park, 20742, MD, USA

**†** These authors contributed equally to this work and share first authorship.

*** Correspondence:** Debabrata Biswas: dbiswas@umd.edu

# Supplementary table

**Table S1. Values calculated from number of isolates co-resistant to two antibiotics, and later used to generate chord plot for analysis and visualization of co-occurrence of antibiotic resistance within farm isolates.**

| **Class of antibiotic 1** | **Antibiotic 1** | **Class of antibiotic 2** | **Antibiotic 2** | **Co-resistant isolates by antibiotic** |
| --- | --- | --- | --- | --- |
| Penicillin | Amoxicillin | Aminoglycoside | Gentamcin | 8 |
|  | Amoxicillin |  | Kanamycin | 41 |
|  | Amoxicillin |  | Streptomycin | 122 |
|  | Amoxicillin | Cephalosporine | Cephradine | 281 |
|  | Amoxicillin |  | Ceftriaxone | 91 |
|  | Amoxicillin | Folate pathway inhibitor | Trimethoprim-Sulfamethoxazole | 76 |
|  | Amoxicillin | Macrolide | Azithromycin | 112 |
|  | Amoxicillin | Phenolic | Chloramphenicol | 68 |
|  | Amoxicillin | Quinolone | Ciprofloxacin | 63 |
|  | Amoxicillin | Tetracycline | Tetracycline | 99 |
|  | Amoxicillin |  | Oxytetracycline | 95 |
|  | Ampicillin | Aminoglycoside | Gentamcin | 9 |
|  | Ampicillin |  | Kanamycin | 37 |
|  | Ampicillin |  | Streptomycin | 132 |
|  | Ampicillin | Cephalosporine | Cephradine | 268 |
|  | Ampicillin |  | Ceftriaxone | 98 |
|  | Ampicillin | Folate pathway inhibitor | Trimethoprim-Sulfamethoxazole | 77 |
|  | Ampicillin | Macrolide | Azithromycin | 115 |
|  | Ampicillin | Penicillin | Amoxicillin | 278 |
|  | Ampicillin | Phenolic | Chloramphenicol | 65 |
|  | Ampicillin | Quinolone | Ciprofloxacin | 64 |
|  | Ampicillin | Tetracycline | Tetracycline | 103 |
|  | Ampicillin |  | Oxytetracycline | 97 |
| Macrolide | Azithromycin | Aminoglycoside | Gentamcin | 10 |
|  | Azithromycin |  | Kanamycin | 27 |
|  | Azithromycin |  | Streptomycin | 88 |
|  | Azithromycin | Cephalosporine | Cephradine | 112 |
|  | Azithromycin |  | Ceftriaxone | 56 |
|  | Azithromycin | Folate pathway inhibitor | Trimethoprim-Sulfamethoxazole | 67 |
|  | Azithromycin | Phenolic | Chloramphenicol | 48 |
|  | Azithromycin | Quinolone | Ciprofloxacin | 61 |
|  | Azithromycin | Tetracycline | Tetracycline | 91 |
|  | Azithromycin |  | Oxytetracycline | 84 |
| Cephalosporine | Ceftriaxone | Aminoglycoside | Gentamcin | 10 |
|  | Ceftriaxone |  | Kanamycin | 18 |
|  | Ceftriaxone |  | Streptomycin | 74 |
|  | Ceftriaxone | Folate pathway inhibitor | Trimethoprim-Sulfamethoxazole | 51 |
|  | Ceftriaxone | Phenolic | Chloramphenicol | 40 |
|  | Ceftriaxone | Quinolone | Ciprofloxacin | 41 |
|  | Ceftriaxone | Tetracycline | Tetracycline | 64 |
|  | Ceftriaxone |  | Oxytetracycline | 61 |
|  | Cephradine | Aminoglycoside | Gentamcin | 14 |
|  | Cephradine |  | Kanamycin | 47 |
|  | Cephradine |  | Streptomycin | 127 |
|  | Cephradine | Cephalosporine | Ceftriaxone | 86 |
|  | Cephradine | Folate pathway inhibitor | Trimethoprim-Sulfamethoxazole | 70 |
|  | Cephradine | Phenolic | Chloramphenicol | 61 |
|  | Cephradine | Quinolone | Ciprofloxacin | 64 |
|  | Cephradine | Tetracycline | Tetracycline | 98 |
|  | Cephradine |  | Oxytetracycline | 91 |
| Phenolic | Chloramphenicol | Aminoglycoside | Gentamcin | 3 |
|  | Chloramphenicol |  | Kanamycin | 10 |
|  | Chloramphenicol |  | Streptomycin | 41 |
|  | Chloramphenicol | Folate pathway inhibitor | Trimethoprim-Sulfamethoxazole | 40 |
|  | Chloramphenicol | Quinolone | Ciprofloxacin | 32 |
|  | Chloramphenicol | Tetracycline | Tetracycline | 47 |
|  | Chloramphenicol |  | Oxytetracycline | 45 |
| Quinolone | Ciprofloxacin | Aminoglycoside | Gentamcin | 12 |
|  | Ciprofloxacin |  | Kanamycin | 24 |
|  | Ciprofloxacin |  | Streptomycin | 64 |
|  | Ciprofloxacin | Folate pathway inhibitor | Trimethoprim-Sulfamethoxazole | 46 |
|  | Ciprofloxacin | Tetracycline | Tetracycline | 56 |
|  | Ciprofloxacin |  | Oxytetracycline | 50 |
| Aminoglycoside | Gentamcin | Aminoglycoside | Kanamycin | 10 |
|  | Gentamcin |  | Streptomycin | 15 |
|  | Gentamcin | Folate pathway inhibitor | Trimethoprim-Sulfamethoxazole | 5 |
|  | Gentamcin | Tetracycline | Tetracycline | 11 |
|  | Gentamcin |  | Oxytetracycline | 10 |
|  | Kanamycin | Aminoglycoside | Streptomycin | 30 |
|  | Kanamycin | Folate pathway inhibitor | Trimethoprim-Sulfamethoxazole | 17 |
|  | Kanamycin | Tetracycline | Tetracycline | 27 |
|  | Kanamycin |  | Oxytetracycline | 24 |
| Tetracycline | Oxytetracycline | Aminoglycoside | Streptomycin | 75 |
|  | Oxytetracycline | Folate pathway inhibitor | Trimethoprim-Sulfamethoxazole | 65 |
|  | Tetracycline | Aminoglycoside | Streptomycin | 79 |
|  | Tetracycline | Folate pathway inhibitor | Trimethoprim-Sulfamethoxazole | 67 |
|  | Tetracycline | Tetracycline | Oxytetracycline | 97 |
| Folate Pathway Inhibitors | Trimethoprim-Sulfamethoxazole | Aminoglycoside | Streptomycin | 66 |
